# Wearing Flannel

**Published:** 1878-09

**Authors:** 


PUT it on at once. Winter or summer, nothing better can be worn next the skin than a loose, red, woollen flannel shirt; "loose," for it has room to move on the skin, thus causing a titilation which draws the blood to the surface and keeps it there; and when that is the case no one can take a cold; "red" for white flannel fulls up, mats together, and becomes tight, stiff, heavy and impervious, "woollen," the product of a sheep, and not of a gentleman of color, not of cotton wool, because that merely *absorbs* the moisture from the surface, while woollen flannel conveys it from the skin and deposites it in drops on the outside of the shirt, from which the ordinary cotton shirt absorbs it, and by its nearer exposure to the exterior air, it is soon dried without injury to the body. Having these properties, red woollen flannel is worn by sailors even in the mid summer of the hottest countries. Wear a thinner material in summer.

